# Supplementary material for: Resolving the full spectrum of human genome variation using Linked-Reads
Source: Genome Res. 2019 Apr;29(4):635–45. doi: 10.1101/gr.234443.118 (PMC6442396; doi:10.1101/gr.234443.118)
Supplement: Supplemental Material [file supp_gr.234443.118_Supplemental_longranger-2.2.zip › longranger-2.2.tar.bz2!/lr2.2-public/lib/go/src/code.google.com/vitess/docs/reference/vtctl.html]

Vitess / vtctl Reference


Toggle navigation

- Overview
- Guides
- Reference
- Blog
- GitHub

- #### Overview

  - What is Vitess
  - Scaling MySQL with Vitess
  - Key Concepts
- #### Getting Started

  - Run Vitess on Kubernetes
    - Custom Docker Build
  - Run Vitess Locally
- #### User Guide

  - Introduction- Client Libraries- Backing Up Data- Reparenting
        - Schema Management
        - VSchema Guide
        - Schema Swap (Tutorial)
        - Sharding
          - Horizontal Sharding (Tutorial, automated)
          - Horizontal Sharding (Tutorial, manual)
          - Sharding in Kubernetes (Tutorial, automated)
          - Sharding in Kubernetes (Tutorial, manual)
        - Vitess Sequences
        - MySQL Server Protocol
        - Vitess and Replication
        - Update Stream
        - Row Based Replication
        - Topology Service
        - Transport Security Model
        - Launching
          - Scalability Philosophy
          - Production Planning
          - Server Configuration
          - 2PC Guide
          - Troubleshooting
        - Upgrading
- #### Reference Guides

  - Vitess API- vtctl Commands
- #### Other Resources

  - Presentations- Blog- Roadmap
- #### Contributing

  - Contributing to Vitess- GitHub Workflow- Code Reviews
- GitHub

# vtctl Reference

- #### Overview

  - What is Vitess
  - Scaling MySQL with Vitess
  - Key Concepts
- #### Getting Started

  - Run Vitess on Kubernetes
    - Custom Docker Build
  - Run Vitess Locally
- #### User Guide

  - Introduction- Client Libraries- Backing Up Data- Reparenting
        - Schema Management
        - VSchema Guide
        - Schema Swap (Tutorial)
        - Sharding
          - Horizontal Sharding (Tutorial, automated)
          - Horizontal Sharding (Tutorial, manual)
          - Sharding in Kubernetes (Tutorial, automated)
          - Sharding in Kubernetes (Tutorial, manual)
        - Vitess Sequences
        - MySQL Server Protocol
        - Vitess and Replication
        - Update Stream
        - Row Based Replication
        - Topology Service
        - Transport Security Model
        - Launching
          - Scalability Philosophy
          - Production Planning
          - Server Configuration
          - 2PC Guide
          - Troubleshooting
        - Upgrading
- #### Reference Guides

  - Vitess API- vtctl Commands
- #### Other Resources

  - Presentations- Blog- Roadmap
- #### Contributing

  - Contributing to Vitess- GitHub Workflow- Code Reviews

This reference guide explains the commands that the **vtctl** tool supports. **vtctl** is a command-line tool used to administer a Vitess cluster, and it allows a human or application to easily interact with a Vitess implementation.

Commands are listed in the following groups:

- Cells
- Generic
- Keyspaces
- Queries
- Replication Graph
- Resharding Throttler
- Schema, Version, Permissions
- Serving Graph
- Shards
- Tablets
- Workflows

## Cells

- AddCellInfo
- DeleteCellInfo
- GetCellInfo
- GetCellInfoNames
- UpdateCellInfo

### AddCellInfo

Registers a local topology service in a new cell by creating the CellInfo with the provided parameters. The address will be used to connect to the topology service, and we'll put Vitess data starting at the provided root.

#### Example

```
AddCellInfo [-server_address <addr>] [-root <root>] <cell>
```

#### Flags

| Name | Type | Definition |
| --- | --- | --- |
| root | string | The root path the topology server is using for that cell. |
| server\_address | string | The address the topology server is using for that cell. |

#### Arguments

- `<addr>` – Required.
- `<cell>` – Required. A cell is a location for a service. Generally, a cell resides in only one cluster. In Vitess, the terms "cell" and "data center" are interchangeable. The argument value is a string that does not contain whitespace.

#### Errors

- the `<cell>` argument is required for the `<AddCellInfo>` command This error occurs if the command is not called with exactly one argument.

### DeleteCellInfo

Deletes the CellInfo for the provided cell. The cell cannot be referenced by any Shard record.

#### Example

```
DeleteCellInfo <cell>
```

#### Errors

- the `<cell>` argument is required for the `<DeleteCellInfo>` command This error occurs if the command is not called with exactly one argument.

### GetCellInfo

Prints a JSON representation of the CellInfo for a cell.

#### Example

```
GetCellInfo <cell>
```

#### Errors

- the `<cell>` argument is required for the `<GetCellInfo>` command This error occurs if the command is not called with exactly one argument.

### GetCellInfoNames

Lists all the cells for which we have a CellInfo object, meaning we have a local topology service registered.

#### Example

```
GetCellInfoNames
```

#### Errors

- `<GetCellInfoNames>` command takes no parameter This error occurs if the command is not called with exactly 0 arguments.

### UpdateCellInfo

Updates the content of a CellInfo with the provided parameters. If a value is empty, it is not updated. The CellInfo will be created if it doesn't exist.

#### Example

```
UpdateCellInfo [-server_address <addr>] [-root <root>] <cell>
```

#### Flags

| Name | Type | Definition |
| --- | --- | --- |
| root | string | The root path the topology server is using for that cell. |
| server\_address | string | The address the topology server is using for that cell. |

#### Arguments

- `<addr>` – Required.
- `<cell>` – Required. A cell is a location for a service. Generally, a cell resides in only one cluster. In Vitess, the terms "cell" and "data center" are interchangeable. The argument value is a string that does not contain whitespace.

#### Errors

- the `<cell>` argument is required for the `<UpdateCellInfo>` command This error occurs if the command is not called with exactly one argument.

## Generic

- ListAllTablets
- ListTablets
- PruneActionLogs
- Validate

### ListAllTablets

Lists all tablets in an awk-friendly way.

#### Example

```
ListAllTablets <cell name>
```

#### Arguments

- `<cell name>` – Required. A cell is a location for a service. Generally, a cell resides in only one cluster. In Vitess, the terms "cell" and "data center" are interchangeable. The argument value is a string that does not contain whitespace.

#### Errors

- the `<cell name>` argument is required for the `<ListAllTablets>` command This error occurs if the command is not called with exactly one argument.

### ListTablets

Lists specified tablets in an awk-friendly way.

#### Example

```
ListTablets <tablet alias> ...
```

#### Arguments

- `<tablet alias>` – Required. A Tablet Alias uniquely identifies a vttablet. The argument value is in the format `<cell name>-<uid>`. To specify multiple values for this argument, separate individual values with a space.

#### Errors

- the `<tablet alias>` argument is required for the `<ListTablets>` command This error occurs if the command is not called with at least one argument.

### PruneActionLogs

(requires zktopo.Server)  
  
e.g. PruneActionLogs -keep-count=10 /zk/global/vt/keyspaces/my\_keyspace/shards/0/actionlog  
  
Removes older actionlog entries until at most <count to keep> are left.

#### Example

```
PruneActionLogs [-keep-count=<count to keep>] <zk actionlog path> ...
```

#### Flags

| Name | Type | Definition |
| --- | --- | --- |
| keep-count | Int | count to keep |

#### Arguments

- `<zk actionlog path>` – Required. To specify multiple values for this argument, separate individual values with a space.

#### Errors

- action `<PruneActionLogs>` requires `<zk action log path>` [...] This error occurs if the command is not called with at least one argument.
- `<PruneActionLogs>` requires a zktopo.Server
- some errors occurred, check the log

### Validate

Validates that all nodes reachable from the global replication graph and that all tablets in all discoverable cells are consistent.

#### Example

```
Validate [-ping-tablets]
```

#### Flags

| Name | Type | Definition |
| --- | --- | --- |
| ping-tablets | Boolean | Indicates whether all tablets should be pinged during the validation process |

## Keyspaces

- CreateKeyspace
- DeleteKeyspace
- FindAllShardsInKeyspace
- GetKeyspace
- GetKeyspaces
- MigrateServedFrom
- MigrateServedTypes
- RebuildKeyspaceGraph
- RemoveKeyspaceCell
- SetKeyspaceServedFrom
- SetKeyspaceShardingInfo
- ValidateKeyspace
- WaitForDrain

### CreateKeyspace

Creates the specified keyspace.

#### Example

```
CreateKeyspace [-sharding_column_name=name] [-sharding_column_type=type] [-served_from=tablettype1:ks1,tablettype2,ks2,...] [-force] <keyspace name>
```

#### Flags

| Name | Type | Definition |
| --- | --- | --- |
| force | Boolean | Proceeds even if the keyspace already exists |
| served\_from | string | Specifies a comma-separated list of dbtype:keyspace pairs used to serve traffic |
| sharding\_column\_name | string | Specifies the column to use for sharding operations |
| sharding\_column\_type | string | Specifies the type of the column to use for sharding operations |

#### Arguments

- `<keyspace name>` – Required. The name of a sharded database that contains one or more tables. Vitess distributes keyspace shards into multiple machines and provides an SQL interface to query the data. The argument value must be a string that does not contain whitespace.

#### Errors

- the `<keyspace name>` argument is required for the `<CreateKeyspace>` command This error occurs if the command is not called with exactly one argument.

### DeleteKeyspace

Deletes the specified keyspace. In recursive mode, it also recursively deletes all shards in the keyspace. Otherwise, there must be no shards left in the keyspace.

#### Example

```
DeleteKeyspace [-recursive] <keyspace>
```

#### Flags

| Name | Type | Definition |
| --- | --- | --- |
| recursive | Boolean | Also recursively delete all shards in the keyspace. |

#### Arguments

- `<keyspace>` – Required. The name of a sharded database that contains one or more tables. Vitess distributes keyspace shards into multiple machines and provides an SQL interface to query the data. The argument value must be a string that does not contain whitespace.

#### Errors

- must specify the `<keyspace>` argument for `<DeleteKeyspace>` This error occurs if the command is not called with exactly one argument.

### FindAllShardsInKeyspace

Displays all of the shards in the specified keyspace.

#### Example

```
FindAllShardsInKeyspace <keyspace>
```

#### Arguments

- `<keyspace>` – Required. The name of a sharded database that contains one or more tables. Vitess distributes keyspace shards into multiple machines and provides an SQL interface to query the data. The argument value must be a string that does not contain whitespace.

#### Errors

- the `<keyspace>` argument is required for the `<FindAllShardsInKeyspace>` command This error occurs if the command is not called with exactly one argument.

### GetKeyspace

Outputs a JSON structure that contains information about the Keyspace.

#### Example

```
GetKeyspace <keyspace>
```

#### Arguments

- `<keyspace>` – Required. The name of a sharded database that contains one or more tables. Vitess distributes keyspace shards into multiple machines and provides an SQL interface to query the data. The argument value must be a string that does not contain whitespace.

#### Errors

- the `<keyspace>` argument is required for the `<GetKeyspace>` command This error occurs if the command is not called with exactly one argument.

### GetKeyspaces

Outputs a sorted list of all keyspaces.

### MigrateServedFrom

Makes the <destination keyspace/shard> serve the given type. This command also rebuilds the serving graph.

#### Example

```
MigrateServedFrom [-cells=c1,c2,...] [-reverse] <destination keyspace/shard> <served tablet type>
```

#### Flags

| Name | Type | Definition |
| --- | --- | --- |
| cells | string | Specifies a comma-separated list of cells to update |
| filtered\_replication\_wait\_time | Duration | Specifies the maximum time to wait, in seconds, for filtered replication to catch up on master migrations |
| reverse | Boolean | Moves the served tablet type backward instead of forward. Use in case of trouble |

#### Arguments

- `<destination keyspace/shard>` – Required. The name of a sharded database that contains one or more tables as well as the shard associated with the command. The keyspace must be identified by a string that does not contain whitepace, while the shard is typically identified by a string in the format `<range start>-<range end>`.
- `<served tablet type>` – Required. The vttablet's role. Valid values are:

  - `backup` – A slaved copy of data that is offline to queries other than for backup purposes
  - `batch` – A slaved copy of data for OLAP load patterns (typically for MapReduce jobs)
  - `drained` – A tablet that is reserved for a background process. For example, a tablet used by a vtworker process, where the tablet is likely lagging in replication.
  - `experimental` – A slaved copy of data that is ready but not serving query traffic. The value indicates a special characteristic of the tablet that indicates the tablet should not be considered a potential master. Vitess also does not worry about lag for experimental tablets when reparenting.
  - `master` – A primary copy of data
  - `rdonly` – A slaved copy of data for OLAP load patterns
  - `replica` – A slaved copy of data ready to be promoted to master
  - `restore` – A tablet that is restoring from a snapshot. Typically, this happens at tablet startup, then it goes to its right state.
  - `schema_apply` – A slaved copy of data that had been serving query traffic but that is now applying a schema change. Following the change, the tablet will revert to its serving type.
  - `snapshot_source` – A slaved copy of data where mysqld is **not** running and where Vitess is serving data files to clone slaves. Use this command to enter this mode:

    ```
    vtctl Snapshot -server-mode ...
    ```

    Use this command to exit this mode:

    ```
    vtctl SnapshotSourceEnd ...
    ```
  - `spare` – A slaved copy of data that is ready but not serving query traffic. The data could be a potential master tablet.

#### Errors

- the `<destination keyspace/shard>` and `<served tablet type>` arguments are both required for the `<MigrateServedFrom>` command This error occurs if the command is not called with exactly 2 arguments.

### MigrateServedTypes

Migrates a serving type from the source shard to the shards that it replicates to. This command also rebuilds the serving graph. The <keyspace/shard> argument can specify any of the shards involved in the migration.

#### Example

```
MigrateServedTypes [-cells=c1,c2,...] [-reverse] [-skip-refresh-state] <keyspace/shard> <served tablet type>
```

#### Flags

| Name | Type | Definition |
| --- | --- | --- |
| cells | string | Specifies a comma-separated list of cells to update |
| filtered\_replication\_wait\_time | Duration | Specifies the maximum time to wait, in seconds, for filtered replication to catch up on master migrations |
| reverse | Boolean | Moves the served tablet type backward instead of forward. Use in case of trouble |
| skip-refresh-state | Boolean | Skips refreshing the state of the source tablets after the migration, meaning that the refresh will need to be done manually, replica and rdonly only) |

#### Arguments

- `<keyspace/shard>` – Required. The name of a sharded database that contains one or more tables as well as the shard associated with the command. The keyspace must be identified by a string that does not contain whitepace, while the shard is typically identified by a string in the format `<range start>-<range end>`.
- `<served tablet type>` – Required. The vttablet's role. Valid values are:

  - `backup` – A slaved copy of data that is offline to queries other than for backup purposes
  - `batch` – A slaved copy of data for OLAP load patterns (typically for MapReduce jobs)
  - `drained` – A tablet that is reserved for a background process. For example, a tablet used by a vtworker process, where the tablet is likely lagging in replication.
  - `experimental` – A slaved copy of data that is ready but not serving query traffic. The value indicates a special characteristic of the tablet that indicates the tablet should not be considered a potential master. Vitess also does not worry about lag for experimental tablets when reparenting.
  - `master` – A primary copy of data
  - `rdonly` – A slaved copy of data for OLAP load patterns
  - `replica` – A slaved copy of data ready to be promoted to master
  - `restore` – A tablet that is restoring from a snapshot. Typically, this happens at tablet startup, then it goes to its right state.
  - `schema_apply` – A slaved copy of data that had been serving query traffic but that is now applying a schema change. Following the change, the tablet will revert to its serving type.
  - `snapshot_source` – A slaved copy of data where mysqld is **not** running and where Vitess is serving data files to clone slaves. Use this command to enter this mode:

    ```
    vtctl Snapshot -server-mode ...
    ```

    Use this command to exit this mode:

    ```
    vtctl SnapshotSourceEnd ...
    ```
  - `spare` – A slaved copy of data that is ready but not serving query traffic. The data could be a potential master tablet.

#### Errors

- the `<source keyspace/shard>` and `<served tablet type>` arguments are both required for the `<MigrateServedTypes>` command This error occurs if the command is not called with exactly 2 arguments.
- the `<skip-refresh-state>` flag can only be specified for non-master migrations

### RebuildKeyspaceGraph

Rebuilds the serving data for the keyspace. This command may trigger an update to all connected clients.

#### Example

```
RebuildKeyspaceGraph [-cells=c1,c2,...] <keyspace> ...
```

#### Flags

| Name | Type | Definition |
| --- | --- | --- |
| cells | string | Specifies a comma-separated list of cells to update |

#### Arguments

- `<keyspace>` – Required. The name of a sharded database that contains one or more tables. Vitess distributes keyspace shards into multiple machines and provides an SQL interface to query the data. The argument value must be a string that does not contain whitespace. To specify multiple values for this argument, separate individual values with a space.

#### Errors

- the `<keyspace>` argument must be used to specify at least one keyspace when calling the `<RebuildKeyspaceGraph>` command This error occurs if the command is not called with at least one argument.

### RemoveKeyspaceCell

Removes the cell from the Cells list for all shards in the keyspace.

#### Example

```
RemoveKeyspaceCell [-force] [-recursive] <keyspace> <cell>
```

#### Flags

| Name | Type | Definition |
| --- | --- | --- |
| force | Boolean | Proceeds even if the cell's topology server cannot be reached. The assumption is that you turned down the entire cell, and just need to update the global topo data. |
| recursive | Boolean | Also delete all tablets in that cell belonging to the specified keyspace. |

#### Arguments

- `<keyspace>` – Required. The name of a sharded database that contains one or more tables. Vitess distributes keyspace shards into multiple machines and provides an SQL interface to query the data. The argument value must be a string that does not contain whitespace.
- `<cell>` – Required. A cell is a location for a service. Generally, a cell resides in only one cluster. In Vitess, the terms "cell" and "data center" are interchangeable. The argument value is a string that does not contain whitespace.

#### Errors

- the `<keyspace>` and `<cell>` arguments are required for the `<RemoveKeyspaceCell>` command This error occurs if the command is not called with exactly 2 arguments.

### SetKeyspaceServedFrom

Changes the ServedFromMap manually. This command is intended for emergency fixes. This field is automatically set when you call the *MigrateServedFrom* command. This command does not rebuild the serving graph.

#### Example

```
SetKeyspaceServedFrom [-source=<source keyspace name>] [-remove] [-cells=c1,c2,...] <keyspace name> <tablet type>
```

#### Flags

| Name | Type | Definition |
| --- | --- | --- |
| cells | string | Specifies a comma-separated list of cells to affect |
| remove | Boolean | Indicates whether to add (default) or remove the served from record |
| source | string | Specifies the source keyspace name |

#### Arguments

- `<keyspace name>` – Required. The name of a sharded database that contains one or more tables. Vitess distributes keyspace shards into multiple machines and provides an SQL interface to query the data. The argument value must be a string that does not contain whitespace.
- `<tablet type>` – Required. The vttablet's role. Valid values are:

  - `backup` – A slaved copy of data that is offline to queries other than for backup purposes
  - `batch` – A slaved copy of data for OLAP load patterns (typically for MapReduce jobs)
  - `drained` – A tablet that is reserved for a background process. For example, a tablet used by a vtworker process, where the tablet is likely lagging in replication.
  - `experimental` – A slaved copy of data that is ready but not serving query traffic. The value indicates a special characteristic of the tablet that indicates the tablet should not be considered a potential master. Vitess also does not worry about lag for experimental tablets when reparenting.
  - `master` – A primary copy of data
  - `rdonly` – A slaved copy of data for OLAP load patterns
  - `replica` – A slaved copy of data ready to be promoted to master
  - `restore` – A tablet that is restoring from a snapshot. Typically, this happens at tablet startup, then it goes to its right state.
  - `schema_apply` – A slaved copy of data that had been serving query traffic but that is now applying a schema change. Following the change, the tablet will revert to its serving type.
  - `snapshot_source` – A slaved copy of data where mysqld is **not** running and where Vitess is serving data files to clone slaves. Use this command to enter this mode:

    ```
    vtctl Snapshot -server-mode ...
    ```

    Use this command to exit this mode:

    ```
    vtctl SnapshotSourceEnd ...
    ```
  - `spare` – A slaved copy of data that is ready but not serving query traffic. The data could be a potential master tablet.

#### Errors

- the `<keyspace name>` and `<tablet type>` arguments are required for the `<SetKeyspaceServedFrom>` command This error occurs if the command is not called with exactly 2 arguments.

### SetKeyspaceShardingInfo

Updates the sharding information for a keyspace.

#### Example

```
SetKeyspaceShardingInfo [-force] <keyspace name> [<column name>] [<column type>]
```

#### Flags

| Name | Type | Definition |
| --- | --- | --- |
| force | Boolean | Updates fields even if they are already set. Use caution before calling this command. |

#### Arguments

- `<keyspace name>` – Required. The name of a sharded database that contains one or more tables. Vitess distributes keyspace shards into multiple machines and provides an SQL interface to query the data. The argument value must be a string that does not contain whitespace.
- `<column name>` – Optional.
- `<column type>` – Optional.

#### Errors

- the `<keyspace name>` argument is required for the `<SetKeyspaceShardingInfo>` command. The `<column name>` and `<column type>` arguments are both optional This error occurs if the command is not called with between 1 and 3 arguments.
- both `<column name>` and `<column type>` must be set, or both must be unset

### ValidateKeyspace

Validates that all nodes reachable from the specified keyspace are consistent.

#### Example

```
ValidateKeyspace [-ping-tablets] <keyspace name>
```

#### Flags

| Name | Type | Definition |
| --- | --- | --- |
| ping-tablets | Boolean | Specifies whether all tablets will be pinged during the validation process |

#### Arguments

- `<keyspace name>` – Required. The name of a sharded database that contains one or more tables. Vitess distributes keyspace shards into multiple machines and provides an SQL interface to query the data. The argument value must be a string that does not contain whitespace.

#### Errors

- the `<keyspace name>` argument is required for the `<ValidateKeyspace>` command This error occurs if the command is not called with exactly one argument.

### WaitForDrain

Blocks until no new queries were observed on all tablets with the given tablet type in the specifed keyspace. This can be used as sanity check to ensure that the tablets were drained after running vtctl MigrateServedTypes and vtgate is no longer using them. If -timeout is set, it fails when the timeout is reached.

#### Example

```
WaitForDrain [-timeout <duration>] [-retry_delay <duration>] [-initial_wait <duration>] <keyspace/shard> <served tablet type>
```

#### Flags

| Name | Type | Definition |
| --- | --- | --- |
| cells | string | Specifies a comma-separated list of cells to look for tablets |
| initial\_wait | Duration | Time to wait for all tablets to check in |
| retry\_delay | Duration | Time to wait between two checks |
| timeout | Duration | Timeout after which the command fails |

#### Arguments

- `<keyspace/shard>` – Required. The name of a sharded database that contains one or more tables as well as the shard associated with the command. The keyspace must be identified by a string that does not contain whitepace, while the shard is typically identified by a string in the format `<range start>-<range end>`.
- `<served tablet type>` – Required. The vttablet's role. Valid values are:

  - `backup` – A slaved copy of data that is offline to queries other than for backup purposes
  - `batch` – A slaved copy of data for OLAP load patterns (typically for MapReduce jobs)
  - `drained` – A tablet that is reserved for a background process. For example, a tablet used by a vtworker process, where the tablet is likely lagging in replication.
  - `experimental` – A slaved copy of data that is ready but not serving query traffic. The value indicates a special characteristic of the tablet that indicates the tablet should not be considered a potential master. Vitess also does not worry about lag for experimental tablets when reparenting.
  - `master` – A primary copy of data
  - `rdonly` – A slaved copy of data for OLAP load patterns
  - `replica` – A slaved copy of data ready to be promoted to master
  - `restore` – A tablet that is restoring from a snapshot. Typically, this happens at tablet startup, then it goes to its right state.
  - `schema_apply` – A slaved copy of data that had been serving query traffic but that is now applying a schema change. Following the change, the tablet will revert to its serving type.
  - `snapshot_source` – A slaved copy of data where mysqld is **not** running and where Vitess is serving data files to clone slaves. Use this command to enter this mode:

    ```
    vtctl Snapshot -server-mode ...
    ```

    Use this command to exit this mode:

    ```
    vtctl SnapshotSourceEnd ...
    ```
  - `spare` – A slaved copy of data that is ready but not serving query traffic. The data could be a potential master tablet.

#### Errors

- the `<keyspace/shard>` and `<tablet type>` arguments are both required for the `<WaitForDrain>` command This error occurs if the command is not called with exactly 2 arguments.

## Queries

- VtGateExecute
- VtGateExecuteKeyspaceIds
- VtGateExecuteShards
- VtGateSplitQuery
- VtTabletBegin
- VtTabletCommit
- VtTabletExecute
- VtTabletRollback
- VtTabletStreamHealth
- VtTabletUpdateStream

### VtGateExecute

Executes the given SQL query with the provided bound variables against the vtgate server.

#### Example

```
VtGateExecute -server <vtgate> [-bind_variables <JSON map>] [-connect_timeout <connect timeout>] [-keyspace <default keyspace>] [-tablet_type <tablet type>] [-options <proto text options>] [-json] <sql>
```

#### Flags

| Name | Type | Definition |
| --- | --- | --- |
| connect\_timeout | Duration | Connection timeout for vtgate client |
| json | Boolean | Output JSON instead of human-readable table |
| keyspace | string | default keyspace to use |
| options | string | execute options values as a text encoded proto of the ExecuteOptions structure |
| server | string | VtGate server to connect to |
| tablet\_type | string | tablet type to query |

#### Arguments

- `<vtgate>` – Required.
- `<sql>` – Required.

#### Errors

- the `<sql>` argument is required for the `<VtGateExecute>` command This error occurs if the command is not called with exactly one argument.
- error connecting to vtgate '%v': %v
- Execute failed: %v

### VtGateExecuteKeyspaceIds

Executes the given SQL query with the provided bound variables against the vtgate server. It is routed to the shards that contain the provided keyspace ids.

#### Example

```
VtGateExecuteKeyspaceIds -server <vtgate> -keyspace <keyspace> -keyspace_ids <ks1 in hex>,<k2 in hex>,... [-bind_variables <JSON map>] [-connect_timeout <connect timeout>] [-tablet_type <tablet type>] [-options <proto text options>] [-json] <sql>
```

#### Flags

| Name | Type | Definition |
| --- | --- | --- |
| connect\_timeout | Duration | Connection timeout for vtgate client |
| json | Boolean | Output JSON instead of human-readable table |
| keyspace | string | keyspace to send query to |
| keyspace\_ids | string | comma-separated list of keyspace ids (in hex) that will map into shards to send query to |
| options | string | execute options values as a text encoded proto of the ExecuteOptions structure |
| server | string | VtGate server to connect to |
| tablet\_type | string | tablet type to query |

#### Arguments

- `<vtgate>` – Required.
- `<keyspace>` – Required. The name of a sharded database that contains one or more tables. Vitess distributes keyspace shards into multiple machines and provides an SQL interface to query the data. The argument value must be a string that does not contain whitespace.
- `<ks1 in hex>` – Required. To specify multiple values for this argument, separate individual values with a comma.
- `<sql>` – Required.

#### Errors

- the `<sql>` argument is required for the `<VtGateExecuteKeyspaceIds>` command This error occurs if the command is not called with exactly one argument.
- cannot hex-decode value %v '%v': %v
- error connecting to vtgate '%v': %v
- Execute failed: %v

### VtGateExecuteShards

Executes the given SQL query with the provided bound variables against the vtgate server. It is routed to the provided shards.

#### Example

```
VtGateExecuteShards -server <vtgate> -keyspace <keyspace> -shards <shard0>,<shard1>,... [-bind_variables <JSON map>] [-connect_timeout <connect timeout>] [-tablet_type <tablet type>] [-options <proto text options>] [-json] <sql>
```

#### Flags

| Name | Type | Definition |
| --- | --- | --- |
| connect\_timeout | Duration | Connection timeout for vtgate client |
| json | Boolean | Output JSON instead of human-readable table |
| keyspace | string | keyspace to send query to |
| options | string | execute options values as a text encoded proto of the ExecuteOptions structure |
| server | string | VtGate server to connect to |
| shards | string | comma-separated list of shards to send query to |
| tablet\_type | string | tablet type to query |

#### Arguments

- `<vtgate>` – Required.
- `<keyspace>` – Required. The name of a sharded database that contains one or more tables. Vitess distributes keyspace shards into multiple machines and provides an SQL interface to query the data. The argument value must be a string that does not contain whitespace.
- `<shard>` – Required. The name of a shard. The argument value is typically in the format `<range start>-<range end>`. To specify multiple values for this argument, separate individual values with a comma.
- `<sql>` – Required.

#### Errors

- the `<sql>` argument is required for the `<VtGateExecuteShards>` command This error occurs if the command is not called with exactly one argument.
- error connecting to vtgate '%v': %v
- Execute failed: %v

### VtGateSplitQuery

Executes the SplitQuery computation for the given SQL query with the provided bound variables against the vtgate server (this is the base query for Map-Reduce workloads, and is provided here for debug / test purposes).

#### Example

```
VtGateSplitQuery -server <vtgate> -keyspace <keyspace> [-split_column <split_column>] -split_count <split_count> [-bind_variables <JSON map>] [-connect_timeout <connect timeout>] <sql>
```

#### Flags

| Name | Type | Definition |
| --- | --- | --- |
| algorithm | string | The algorithm to |
| connect\_timeout | Duration | Connection timeout for vtgate client |
| keyspace | string | keyspace to send query to |
| server | string | VtGate server to connect to |
| split\_count | Int64 | number of splits to generate. |

#### Arguments

- `<vtgate>` – Required.
- `<keyspace>` – Required. The name of a sharded database that contains one or more tables. Vitess distributes keyspace shards into multiple machines and provides an SQL interface to query the data. The argument value must be a string that does not contain whitespace.
- `<split_count>` – Required.
- `<sql>` – Required.

#### Errors

- the `<sql>` argument is required for the `<VtGateSplitQuery>` command This error occurs if the command is not called with exactly one argument.
- Exactly one of `<split_count>` or num\_rows\_per\_query\_part
- Unknown split-query `<algorithm>`: %v
- error connecting to vtgate '%v': %v
- SplitQuery failed: %v

### VtTabletBegin

Starts a transaction on the provided server.

#### Example

```
VtTabletBegin [-username <TableACL user>] [-connect_timeout <connect timeout>] <tablet alias>
```

#### Flags

| Name | Type | Definition |
| --- | --- | --- |
| connect\_timeout | Duration | Connection timeout for vttablet client |
| username | string | If set, value is set as immediate caller id in the request and used by vttablet for TableACL check |

#### Arguments

- `<TableACL user>` – Required.
- `<tablet alias>` – Required. A Tablet Alias uniquely identifies a vttablet. The argument value is in the format `<cell name>-<uid>`.

#### Errors

- the `<tablet_alias>` argument is required for the `<VtTabletBegin>` command This error occurs if the command is not called with exactly one argument.
- cannot connect to tablet %v: %v
- Begin failed: %v

### VtTabletCommit

Commits the given transaction on the provided server.

#### Example

```
VtTabletCommit [-username <TableACL user>] [-connect_timeout <connect timeout>] <transaction_id>
```

#### Flags

| Name | Type | Definition |
| --- | --- | --- |
| connect\_timeout | Duration | Connection timeout for vttablet client |
| username | string | If set, value is set as immediate caller id in the request and used by vttablet for TableACL check |

#### Arguments

- `<TableACL user>` – Required.
- `<transaction_id>` – Required.

#### Errors

- the `<tablet_alias>` and `<transaction_id>` arguments are required for the `<VtTabletCommit>` command This error occurs if the command is not called with exactly 2 arguments.
- cannot connect to tablet %v: %v

### VtTabletExecute

Executes the given query on the given tablet. -transaction\_id is optional. Use VtTabletBegin to start a transaction.

#### Example

```
VtTabletExecute [-username <TableACL user>] [-connect_timeout <connect timeout>] [-transaction_id <transaction_id>] [-options <proto text options>] [-json] <tablet alias> <sql>
```

#### Flags

| Name | Type | Definition |
| --- | --- | --- |
| connect\_timeout | Duration | Connection timeout for vttablet client |
| json | Boolean | Output JSON instead of human-readable table |
| options | string | execute options values as a text encoded proto of the ExecuteOptions structure |
| transaction\_id | Int | transaction id to use, if inside a transaction. |
| username | string | If set, value is set as immediate caller id in the request and used by vttablet for TableACL check |

#### Arguments

- `<TableACL user>` – Required.
- `<tablet alias>` – Required. A Tablet Alias uniquely identifies a vttablet. The argument value is in the format `<cell name>-<uid>`.
- `<sql>` – Required.

#### Errors

- the `<tablet_alias>` and `<sql>` arguments are required for the `<VtTabletExecute>` command This error occurs if the command is not called with exactly 2 arguments.
- cannot connect to tablet %v: %v
- Execute failed: %v

### VtTabletRollback

Rollbacks the given transaction on the provided server.

#### Example

```
VtTabletRollback [-username <TableACL user>] [-connect_timeout <connect timeout>] <tablet alias> <transaction_id>
```

#### Flags

| Name | Type | Definition |
| --- | --- | --- |
| connect\_timeout | Duration | Connection timeout for vttablet client |
| username | string | If set, value is set as immediate caller id in the request and used by vttablet for TableACL check |

#### Arguments

- `<TableACL user>` – Required.
- `<tablet alias>` – Required. A Tablet Alias uniquely identifies a vttablet. The argument value is in the format `<cell name>-<uid>`.
- `<transaction_id>` – Required.

#### Errors

- the `<tablet_alias>` and `<transaction_id>` arguments are required for the `<VtTabletRollback>` command This error occurs if the command is not called with exactly 2 arguments.
- cannot connect to tablet %v: %v

### VtTabletStreamHealth

Executes the StreamHealth streaming query to a vttablet process. Will stop after getting <count> answers.

#### Example

```
VtTabletStreamHealth [-count <count, default 1>] [-connect_timeout <connect timeout>] <tablet alias>
```

#### Flags

| Name | Type | Definition |
| --- | --- | --- |
| connect\_timeout | Duration | Connection timeout for vttablet client |
| count | Int | number of responses to wait for |

#### Arguments

- `<count default 1>` – Required.
- `<tablet alias>` – Required. A Tablet Alias uniquely identifies a vttablet. The argument value is in the format `<cell name>-<uid>`.

#### Errors

- the `<tablet alias>` argument is required for the `<VtTabletStreamHealth>` command This error occurs if the command is not called with exactly one argument.
- cannot connect to tablet %v: %v

### VtTabletUpdateStream

Executes the UpdateStream streaming query to a vttablet process. Will stop after getting <count> answers.

#### Example

```
VtTabletUpdateStream [-count <count, default 1>] [-connect_timeout <connect timeout>] [-position <position>] [-timestamp <timestamp>] <tablet alias>
```

#### Flags

| Name | Type | Definition |
| --- | --- | --- |
| connect\_timeout | Duration | Connection timeout for vttablet client |
| count | Int | number of responses to wait for |
| position | string | position to start the stream from |
| timestamp | Int | timestamp to start the stream from |

#### Arguments

- `<count default 1>` – Required.
- `<tablet alias>` – Required. A Tablet Alias uniquely identifies a vttablet. The argument value is in the format `<cell name>-<uid>`.

#### Errors

- the `<tablet alias>` argument is required for the `<VtTabletUpdateStream>` command This error occurs if the command is not called with exactly one argument.
- cannot connect to tablet %v: %v

## Replication Graph

- GetShardReplication

### GetShardReplication

Outputs a JSON structure that contains information about the ShardReplication.

#### Example

```
GetShardReplication <cell> <keyspace/shard>
```

#### Arguments

- `<cell>` – Required. A cell is a location for a service. Generally, a cell resides in only one cluster. In Vitess, the terms "cell" and "data center" are interchangeable. The argument value is a string that does not contain whitespace.
- `<keyspace/shard>` – Required. The name of a sharded database that contains one or more tables as well as the shard associated with the command. The keyspace must be identified by a string that does not contain whitepace, while the shard is typically identified by a string in the format `<range start>-<range end>`.

#### Errors

- the `<cell>` and `<keyspace/shard>` arguments are required for the `<GetShardReplication>` command This error occurs if the command is not called with exactly 2 arguments.

## Resharding Throttler

- GetThrottlerConfiguration
- ResetThrottlerConfiguration
- ThrottlerMaxRates
- ThrottlerSetMaxRate
- UpdateThrottlerConfiguration

### GetThrottlerConfiguration

Returns the current configuration of the MaxReplicationLag module. If no throttler name is specified, the configuration of all throttlers will be returned.

#### Example

```
GetThrottlerConfiguration -server <vtworker or vttablet> [<throttler name>]
```

#### Flags

| Name | Type | Definition |
| --- | --- | --- |
| server | string | vtworker or vttablet to connect to |

#### Arguments

- `<vtworker or vttablet>` – Required.
- `<throttler name>` – Optional.

#### Errors

- the `<GetThrottlerConfiguration>` command accepts only `<throttler name>` as optional positional parameter This error occurs if the command is not called with more than 1 arguments.
- error creating a throttler client for `<server>` '%v': %v
- failed to get the throttler configuration from `<server>` '%v': %v

### ResetThrottlerConfiguration

Resets the current configuration of the MaxReplicationLag module. If no throttler name is specified, the configuration of all throttlers will be reset.

#### Example

```
ResetThrottlerConfiguration -server <vtworker or vttablet> [<throttler name>]
```

#### Flags

| Name | Type | Definition |
| --- | --- | --- |
| server | string | vtworker or vttablet to connect to |

#### Arguments

- `<vtworker or vttablet>` – Required.
- `<throttler name>` – Optional.

#### Errors

- the `<ResetThrottlerConfiguration>` command accepts only `<throttler name>` as optional positional parameter This error occurs if the command is not called with more than 1 arguments.
- error creating a throttler client for `<server>` '%v': %v
- failed to get the throttler configuration from `<server>` '%v': %v

### ThrottlerMaxRates

Returns the current max rate of all active resharding throttlers on the server.

#### Example

```
ThrottlerMaxRates -server <vtworker or vttablet>
```

#### Flags

| Name | Type | Definition |
| --- | --- | --- |
| server | string | vtworker or vttablet to connect to |

#### Arguments

- `<vtworker or vttablet>` – Required.

#### Errors

- the ThrottlerSetMaxRate command does not accept any positional parameters This error occurs if the command is not called with exactly 0 arguments.
- error creating a throttler client for `<server>` '%v': %v
- failed to get the throttler rate from `<server>` '%v': %v

### ThrottlerSetMaxRate

Sets the max rate for all active resharding throttlers on the server.

#### Example

```
ThrottlerSetMaxRate -server <vtworker or vttablet> <rate>
```

#### Flags

| Name | Type | Definition |
| --- | --- | --- |
| server | string | vtworker or vttablet to connect to |

#### Arguments

- `<vtworker or vttablet>` – Required.
- `<rate>` – Required.

#### Errors

- the `<rate>` argument is required for the `<ThrottlerSetMaxRate>` command This error occurs if the command is not called with exactly one argument.
- failed to parse rate '%v' as integer value: %v
- error creating a throttler client for `<server>` '%v': %v
- failed to set the throttler rate on `<server>` '%v': %v

### UpdateThrottlerConfiguration

Updates the configuration of the MaxReplicationLag module. The configuration must be specified as protobuf text. If a field is omitted or has a zero value, it will be ignored unless -copy\_zero\_values is specified. If no throttler name is specified, all throttlers will be updated.

#### Example

```
UpdateThrottlerConfiguration `-server <vtworker or vttablet> [-copy_zero_values] "<configuration protobuf text>" [<throttler name>]`
```

#### Flags

| Name | Type | Definition |
| --- | --- | --- |
| copy\_zero\_values | Boolean | If true, fields with zero values will be copied as well |
| server | string | vtworker or vttablet to connect to |

#### Arguments

- `<vtworker or vttablet>` – Required.
- `<throttler name>` – Optional.

#### Errors

- Failed to unmarshal the configuration protobuf text (%v) into a protobuf instance: %v
- error creating a throttler client for `<server>` '%v': %v
- failed to update the throttler configuration on `<server>` '%v': %v

## Schema, Version, Permissions

- ApplySchema
- ApplyVSchema
- CopySchemaShard
- GetPermissions
- GetSchema
- GetVSchema
- RebuildVSchemaGraph
- ReloadSchema
- ReloadSchemaKeyspace
- ReloadSchemaShard
- ValidatePermissionsKeyspace
- ValidatePermissionsShard
- ValidateSchemaKeyspace
- ValidateSchemaShard
- ValidateVersionKeyspace
- ValidateVersionShard

### ApplySchema

Applies the schema change to the specified keyspace on every master, running in parallel on all shards. The changes are then propagated to slaves via replication. If -allow\_long\_unavailability is set, schema changes affecting a large number of rows (and possibly incurring a longer period of unavailability) will not be rejected.

#### Example

```
ApplySchema [-allow_long_unavailability] [-wait_slave_timeout=10s] {-sql=<sql> || -sql-file=<filename>} <keyspace>
```

#### Flags

| Name | Type | Definition |
| --- | --- | --- |
| allow\_long\_unavailability | Boolean | Allow large schema changes which incur a longer unavailability of the database. |
| sql | string | A list of semicolon-delimited SQL commands |
| sql-file | string | Identifies the file that contains the SQL commands |
| wait\_slave\_timeout | Duration | The amount of time to wait for slaves to receive the schema change via replication. |

#### Arguments

- `<keyspace>` – Required. The name of a sharded database that contains one or more tables. Vitess distributes keyspace shards into multiple machines and provides an SQL interface to query the data. The argument value must be a string that does not contain whitespace.

#### Errors

- the `<keyspace>` argument is required for the command`<ApplySchema>` command This error occurs if the command is not called with exactly one argument.

### ApplyVSchema

Applies the VTGate routing schema to the provided keyspace. Shows the result after application.

#### Example

```
ApplyVSchema {-vschema=<vschema> || -vschema_file=<vschema file>} [-cells=c1,c2,...] [-skip_rebuild] <keyspace>
```

#### Flags

| Name | Type | Definition |
| --- | --- | --- |
| cells | string | If specified, limits the rebuild to the cells, after upload. Ignored if skipRebuild is set. |
| skip\_rebuild | Boolean | If set, do no rebuild the SrvSchema objects. |
| vschema | string | Identifies the VTGate routing schema |
| vschema\_file | string | Identifies the VTGate routing schema file |

#### Arguments

- `<keyspace>` – Required. The name of a sharded database that contains one or more tables. Vitess distributes keyspace shards into multiple machines and provides an SQL interface to query the data. The argument value must be a string that does not contain whitespace.

#### Errors

- the `<keyspace>` argument is required for the `<ApplyVSchema>` command This error occurs if the command is not called with exactly one argument.
- either the `<vschema>` or `<vschema>`File flag must be specified when calling the `<ApplyVSchema>` command

### CopySchemaShard

Copies the schema from a source shard's master (or a specific tablet) to a destination shard. The schema is applied directly on the master of the destination shard, and it is propagated to the replicas through binlogs.

#### Example

```
CopySchemaShard [-tables=<table1>,<table2>,...] [-exclude_tables=<table1>,<table2>,...] [-include-views] [-wait_slave_timeout=10s] {<source keyspace/shard> || <source tablet alias>} <destination keyspace/shard>
```

#### Flags

| Name | Type | Definition |
| --- | --- | --- |
| exclude\_tables | string | Specifies a comma-separated list of tables to exclude. Each is either an exact match, or a regular expression of the form /regexp/ |
| include-views | Boolean | Includes views in the output |
| tables | string | Specifies a comma-separated list of tables to copy. Each is either an exact match, or a regular expression of the form /regexp/ |
| wait\_slave\_timeout | Duration | The amount of time to wait for slaves to receive the schema change via replication. |

#### Arguments

- `<source tablet alias>` – Required. A Tablet Alias uniquely identifies a vttablet. The argument value is in the format `<cell name>-<uid>`.
- `<destination keyspace/shard>` – Required. The name of a sharded database that contains one or more tables as well as the shard associated with the command. The keyspace must be identified by a string that does not contain whitepace, while the shard is typically identified by a string in the format `<range start>-<range end>`.

#### Errors

- the `<source keyspace/shard>` and `<destination keyspace/shard>` arguments are both required for the `<CopySchemaShard>` command. Instead of the `<source keyspace/shard>` argument, you can also specify `<tablet alias>` which refers to a specific tablet of the shard in the source keyspace This error occurs if the command is not called with exactly 2 arguments.

### GetPermissions

Displays the permissions for a tablet.

#### Example

```
GetPermissions <tablet alias>
```

#### Arguments

- `<tablet alias>` – Required. A Tablet Alias uniquely identifies a vttablet. The argument value is in the format `<cell name>-<uid>`.

#### Errors

- the `<tablet alias>` argument is required for the `<GetPermissions>` command This error occurs if the command is not called with exactly one argument.

### GetSchema

Displays the full schema for a tablet, or just the schema for the specified tables in that tablet.

#### Example

```
GetSchema [-tables=<table1>,<table2>,...] [-exclude_tables=<table1>,<table2>,...] [-include-views] <tablet alias>
```

#### Flags

| Name | Type | Definition |
| --- | --- | --- |
| exclude\_tables | string | Specifies a comma-separated list of tables to exclude. Each is either an exact match, or a regular expression of the form /regexp/ |
| include-views | Boolean | Includes views in the output |
| table\_names\_only | Boolean | Only displays table names that match |
| tables | string | Specifies a comma-separated list of tables for which we should gather information. Each is either an exact match, or a regular expression of the form /regexp/ |

#### Arguments

- `<tablet alias>` – Required. A Tablet Alias uniquely identifies a vttablet. The argument value is in the format `<cell name>-<uid>`.

#### Errors

- the `<tablet alias>` argument is required for the `<GetSchema>` command This error occurs if the command is not called with exactly one argument.

### GetVSchema

Displays the VTGate routing schema.

#### Example

```
GetVSchema <keyspace>
```

#### Arguments

- `<keyspace>` – Required. The name of a sharded database that contains one or more tables. Vitess distributes keyspace shards into multiple machines and provides an SQL interface to query the data. The argument value must be a string that does not contain whitespace.

#### Errors

- the `<keyspace>` argument is required for the `<GetVSchema>` command This error occurs if the command is not called with exactly one argument.

### RebuildVSchemaGraph

Rebuilds the cell-specific SrvVSchema from the global VSchema objects in the provided cells (or all cells if none provided).

#### Example

```
RebuildVSchemaGraph [-cells=c1,c2,...]
```

#### Flags

| Name | Type | Definition |
| --- | --- | --- |
| cells | string | Specifies a comma-separated list of cells to look for tablets |

#### Errors

- `<RebuildVSchemaGraph>` doesn't take any arguments This error occurs if the command is not called with exactly 0 arguments.

### ReloadSchema

Reloads the schema on a remote tablet.

#### Example

```
ReloadSchema <tablet alias>
```

#### Arguments

- `<tablet alias>` – Required. A Tablet Alias uniquely identifies a vttablet. The argument value is in the format `<cell name>-<uid>`.

#### Errors

- the `<tablet alias>` argument is required for the `<ReloadSchema>` command This error occurs if the command is not called with exactly one argument.

### ReloadSchemaKeyspace

Reloads the schema on all the tablets in a keyspace.

#### Example

```
ReloadSchemaKeyspace [-concurrency=10] [-include_master=false] <keyspace>
```

#### Flags

| Name | Type | Definition |
| --- | --- | --- |
| concurrency | Int | How many tablets to reload in parallel |
| include\_master | Boolean | Include the master tablet(s) |

#### Arguments

- `<keyspace>` – Required. The name of a sharded database that contains one or more tables. Vitess distributes keyspace shards into multiple machines and provides an SQL interface to query the data. The argument value must be a string that does not contain whitespace.

#### Errors

- the `<keyspace>` argument is required for the `<ReloadSchemaKeyspace>` command This error occurs if the command is not called with exactly one argument.

### ReloadSchemaShard

Reloads the schema on all the tablets in a shard.

#### Example

```
ReloadSchemaShard [-concurrency=10] [-include_master=false] <keyspace/shard>
```

#### Flags

| Name | Type | Definition |
| --- | --- | --- |
| concurrency | Int | How many tablets to reload in parallel |
| include\_master | Boolean | Include the master tablet |

#### Arguments

- `<keyspace/shard>` – Required. The name of a sharded database that contains one or more tables as well as the shard associated with the command. The keyspace must be identified by a string that does not contain whitepace, while the shard is typically identified by a string in the format `<range start>-<range end>`.

#### Errors

- the `<keyspace/shard>` argument is required for the `<ReloadSchemaShard>` command This error occurs if the command is not called with exactly one argument.

### ValidatePermissionsKeyspace

Validates that the master permissions from shard 0 match those of all of the other tablets in the keyspace.

#### Example

```
ValidatePermissionsKeyspace <keyspace name>
```

#### Arguments

- `<keyspace name>` – Required. The name of a sharded database that contains one or more tables. Vitess distributes keyspace shards into multiple machines and provides an SQL interface to query the data. The argument value must be a string that does not contain whitespace.

#### Errors

- the `<keyspace name>` argument is required for the `<ValidatePermissionsKeyspace>` command This error occurs if the command is not called with exactly one argument.

### ValidatePermissionsShard

Validates that the master permissions match all the slaves.

#### Example

```
ValidatePermissionsShard <keyspace/shard>
```

#### Arguments

- `<keyspace/shard>` – Required. The name of a sharded database that contains one or more tables as well as the shard associated with the command. The keyspace must be identified by a string that does not contain whitepace, while the shard is typically identified by a string in the format `<range start>-<range end>`.

#### Errors

- the `<keyspace/shard>` argument is required for the `<ValidatePermissionsShard>` command This error occurs if the command is not called with exactly one argument.

### ValidateSchemaKeyspace

Validates that the master schema from shard 0 matches the schema on all of the other tablets in the keyspace.

#### Example

```
ValidateSchemaKeyspace [-exclude_tables=''] [-include-views] <keyspace name>
```

#### Flags

| Name | Type | Definition |
| --- | --- | --- |
| exclude\_tables | string | Specifies a comma-separated list of tables to exclude. Each is either an exact match, or a regular expression of the form /regexp/ |
| include-views | Boolean | Includes views in the validation |

#### Arguments

- `<keyspace name>` – Required. The name of a sharded database that contains one or more tables. Vitess distributes keyspace shards into multiple machines and provides an SQL interface to query the data. The argument value must be a string that does not contain whitespace.

#### Errors

- the `<keyspace name>` argument is required for the `<ValidateSchemaKeyspace>` command This error occurs if the command is not called with exactly one argument.

### ValidateSchemaShard

Validates that the master schema matches all of the slaves.

#### Example

```
ValidateSchemaShard [-exclude_tables=''] [-include-views] <keyspace/shard>
```

#### Flags

| Name | Type | Definition |
| --- | --- | --- |
| exclude\_tables | string | Specifies a comma-separated list of tables to exclude. Each is either an exact match, or a regular expression of the form /regexp/ |
| include-views | Boolean | Includes views in the validation |

#### Arguments

- `<keyspace/shard>` – Required. The name of a sharded database that contains one or more tables as well as the shard associated with the command. The keyspace must be identified by a string that does not contain whitepace, while the shard is typically identified by a string in the format `<range start>-<range end>`.

#### Errors

- the `<keyspace/shard>` argument is required for the `<ValidateSchemaShard>` command This error occurs if the command is not called with exactly one argument.

### ValidateVersionKeyspace

Validates that the master version from shard 0 matches all of the other tablets in the keyspace.

#### Example

```
ValidateVersionKeyspace <keyspace name>
```

#### Arguments

- `<keyspace name>` – Required. The name of a sharded database that contains one or more tables. Vitess distributes keyspace shards into multiple machines and provides an SQL interface to query the data. The argument value must be a string that does not contain whitespace.

#### Errors

- the `<keyspace name>` argument is required for the `<ValidateVersionKeyspace>` command This error occurs if the command is not called with exactly one argument.

### ValidateVersionShard

Validates that the master version matches all of the slaves.

#### Example

```
ValidateVersionShard <keyspace/shard>
```

#### Arguments

- `<keyspace/shard>` – Required. The name of a sharded database that contains one or more tables as well as the shard associated with the command. The keyspace must be identified by a string that does not contain whitepace, while the shard is typically identified by a string in the format `<range start>-<range end>`.

#### Errors

- the `<keyspace/shard>` argument is required for the `<ValidateVersionShard>` command This error occurs if the command is not called with exactly one argument.

## Serving Graph

- GetSrvKeyspace
- GetSrvKeyspaceNames
- GetSrvVSchema

### GetSrvKeyspace

Outputs a JSON structure that contains information about the SrvKeyspace.

#### Example

```
GetSrvKeyspace <cell> <keyspace>
```

#### Arguments

- `<cell>` – Required. A cell is a location for a service. Generally, a cell resides in only one cluster. In Vitess, the terms "cell" and "data center" are interchangeable. The argument value is a string that does not contain whitespace.
- `<keyspace>` – Required. The name of a sharded database that contains one or more tables. Vitess distributes keyspace shards into multiple machines and provides an SQL interface to query the data. The argument value must be a string that does not contain whitespace.

#### Errors

- the `<cell>` and `<keyspace>` arguments are required for the `<GetSrvKeyspace>` command This error occurs if the command is not called with exactly 2 arguments.

### GetSrvKeyspaceNames

Outputs a list of keyspace names.

#### Example

```
GetSrvKeyspaceNames <cell>
```

#### Arguments

- `<cell>` – Required. A cell is a location for a service. Generally, a cell resides in only one cluster. In Vitess, the terms "cell" and "data center" are interchangeable. The argument value is a string that does not contain whitespace.

#### Errors

- the `<cell>` argument is required for the `<GetSrvKeyspaceNames>` command This error occurs if the command is not called with exactly one argument.

### GetSrvVSchema

Outputs a JSON structure that contains information about the SrvVSchema.

#### Example

```
GetSrvVSchema <cell>
```

#### Arguments

- `<cell>` – Required. A cell is a location for a service. Generally, a cell resides in only one cluster. In Vitess, the terms "cell" and "data center" are interchangeable. The argument value is a string that does not contain whitespace.

#### Errors

- the `<cell>` argument is required for the `<GetSrvVSchema>` command This error occurs if the command is not called with exactly one argument.

## Shards

- CreateShard
- DeleteShard
- EmergencyReparentShard
- GetShard
- InitShardMaster
- ListBackups
- ListShardTablets
- PlannedReparentShard
- RemoveBackup
- RemoveShardCell
- SetShardServedTypes
- SetShardTabletControl
- ShardReplicationFix
- ShardReplicationPositions
- SourceShardAdd
- SourceShardDelete
- TabletExternallyReparented
- ValidateShard
- WaitForFilteredReplication

### CreateShard

Creates the specified shard.

#### Example

```
CreateShard [-force] [-parent] <keyspace/shard>
```

#### Flags

| Name | Type | Definition |
| --- | --- | --- |
| force | Boolean | Proceeds with the command even if the keyspace already exists |
| parent | Boolean | Creates the parent keyspace if it doesn't already exist |

#### Arguments

- `<keyspace/shard>` – Required. The name of a sharded database that contains one or more tables as well as the shard associated with the command. The keyspace must be identified by a string that does not contain whitepace, while the shard is typically identified by a string in the format `<range start>-<range end>`.

#### Errors

- the `<keyspace/shard>` argument is required for the `<CreateShard>` command This error occurs if the command is not called with exactly one argument.

### DeleteShard

Deletes the specified shard(s). In recursive mode, it also deletes all tablets belonging to the shard. Otherwise, there must be no tablets left in the shard.

#### Example

```
DeleteShard [-recursive] [-even_if_serving] <keyspace/shard> ...
```

#### Flags

| Name | Type | Definition |
| --- | --- | --- |
| even\_if\_serving | Boolean | Remove the shard even if it is serving. Use with caution. |
| recursive | Boolean | Also delete all tablets belonging to the shard. |

#### Arguments

- `<keyspace/shard>` – Required. The name of a sharded database that contains one or more tables as well as the shard associated with the command. The keyspace must be identified by a string that does not contain whitepace, while the shard is typically identified by a string in the format `<range start>-<range end>`. To specify multiple values for this argument, separate individual values with a space.

#### Errors

- the `<keyspace/shard>` argument must be used to identify at least one keyspace and shard when calling the `<DeleteShard>` command This error occurs if the command is not called with at least one argument.

### EmergencyReparentShard

Reparents the shard to the new master. Assumes the old master is dead and not responsding.

#### Example

```
EmergencyReparentShard -keyspace_shard=<keyspace/shard> -new_master=<tablet alias>
```

#### Flags

| Name | Type | Definition |
| --- | --- | --- |
| keyspace\_shard | string | keyspace/shard of the shard that needs to be reparented |
| new\_master | string | alias of a tablet that should be the new master |
| wait\_slave\_timeout | Duration | time to wait for slaves to catch up in reparenting |

#### Errors

- action `<EmergencyReparentShard>` requires -keyspace\_shard=`<keyspace/shard>` -new\_master=`<tablet alias>` This error occurs if the command is not called with exactly 0 arguments.
- cannot use legacy syntax and flag -`<new_master>` for action `<EmergencyReparentShard>` at the same time

### GetShard

Outputs a JSON structure that contains information about the Shard.

#### Example

```
GetShard <keyspace/shard>
```

#### Arguments

- `<keyspace/shard>` – Required. The name of a sharded database that contains one or more tables as well as the shard associated with the command. The keyspace must be identified by a string that does not contain whitepace, while the shard is typically identified by a string in the format `<range start>-<range end>`.

#### Errors

- the `<keyspace/shard>` argument is required for the `<GetShard>` command This error occurs if the command is not called with exactly one argument.

### InitShardMaster

Sets the initial master for a shard. Will make all other tablets in the shard slaves of the provided master. WARNING: this could cause data loss on an already replicating shard. PlannedReparentShard or EmergencyReparentShard should be used instead.

#### Example

```
InitShardMaster [-force] [-wait_slave_timeout=<duration>] <keyspace/shard> <tablet alias>
```

#### Flags

| Name | Type | Definition |
| --- | --- | --- |
| force | Boolean | will force the reparent even if the provided tablet is not a master or the shard master |
| wait\_slave\_timeout | Duration | time to wait for slaves to catch up in reparenting |

#### Arguments

- `<keyspace/shard>` – Required. The name of a sharded database that contains one or more tables as well as the shard associated with the command. The keyspace must be identified by a string that does not contain whitepace, while the shard is typically identified by a string in the format `<range start>-<range end>`.
- `<tablet alias>` – Required. A Tablet Alias uniquely identifies a vttablet. The argument value is in the format `<cell name>-<uid>`.

#### Errors

- action `<InitShardMaster>` requires `<keyspace/shard>` `<tablet alias>` This error occurs if the command is not called with exactly 2 arguments.

### ListBackups

Lists all the backups for a shard.

#### Example

```
ListBackups <keyspace/shard>
```

#### Errors

- action `<ListBackups>` requires `<keyspace/shard>` This error occurs if the command is not called with exactly one argument.

### ListShardTablets

Lists all tablets in the specified shard.

#### Example

```
ListShardTablets <keyspace/shard>
```

#### Arguments

- `<keyspace/shard>` – Required. The name of a sharded database that contains one or more tables as well as the shard associated with the command. The keyspace must be identified by a string that does not contain whitepace, while the shard is typically identified by a string in the format `<range start>-<range end>`.

#### Errors

- the `<keyspace/shard>` argument is required for the `<ListShardTablets>` command This error occurs if the command is not called with exactly one argument.

### PlannedReparentShard

Reparents the shard to the new master, or away from old master. Both old and new master need to be up and running.

#### Example

```
PlannedReparentShard -keyspace_shard=<keyspace/shard> [-new_master=<tablet alias>] [-avoid_master=<tablet alias>]
```

#### Flags

| Name | Type | Definition |
| --- | --- | --- |
| avoid\_master | string | alias of a tablet that should not be the master, i.e. reparent to any other tablet if this one is the master |
| keyspace\_shard | string | keyspace/shard of the shard that needs to be reparented |
| new\_master | string | alias of a tablet that should be the new master |
| wait\_slave\_timeout | Duration | time to wait for slaves to catch up in reparenting |

#### Errors

- action `<PlannedReparentShard>` requires -keyspace\_shard=`<keyspace/shard>` [-new\_master=`<tablet alias>`] [-avoid\_master=`<tablet alias>`] This error occurs if the command is not called with exactly 0 arguments.
- cannot use legacy syntax and flags -`<keyspace_shard>` and -`<new_master>` for action `<PlannedReparentShard>` at the same time

### RemoveBackup

Removes a backup for the BackupStorage.

#### Example

```
RemoveBackup <keyspace/shard> <backup name>
```

#### Arguments

- `<backup name>` – Required.

#### Errors

- action `<RemoveBackup>` requires `<keyspace/shard>` `<backup name>` This error occurs if the command is not called with exactly 2 arguments.

### RemoveShardCell

Removes the cell from the shard's Cells list.

#### Example

```
RemoveShardCell [-force] [-recursive] <keyspace/shard> <cell>
```

#### Flags

| Name | Type | Definition |
| --- | --- | --- |
| force | Boolean | Proceeds even if the cell's topology server cannot be reached. The assumption is that you turned down the entire cell, and just need to update the global topo data. |
| recursive | Boolean | Also delete all tablets in that cell belonging to the specified shard. |

#### Arguments

- `<keyspace/shard>` – Required. The name of a sharded database that contains one or more tables as well as the shard associated with the command. The keyspace must be identified by a string that does not contain whitepace, while the shard is typically identified by a string in the format `<range start>-<range end>`.
- `<cell>` – Required. A cell is a location for a service. Generally, a cell resides in only one cluster. In Vitess, the terms "cell" and "data center" are interchangeable. The argument value is a string that does not contain whitespace.

#### Errors

- the `<keyspace/shard>` and `<cell>` arguments are required for the `<RemoveShardCell>` command This error occurs if the command is not called with exactly 2 arguments.

### SetShardServedTypes

Add or remove served type to/from a shard. This is meant as an emergency function. It does not rebuild any serving graph i.e. does not run 'RebuildKeyspaceGraph'.

#### Example

```
SetShardServedTypes [--cells=c1,c2,...] [--remove] <keyspace/shard> <served tablet type>
```

#### Flags

| Name | Type | Definition |
| --- | --- | --- |
| cells | string | Specifies a comma-separated list of cells to update |
| remove | Boolean | Removes the served tablet type |

#### Arguments

- `<keyspace/shard>` – Required. The name of a sharded database that contains one or more tables as well as the shard associated with the command. The keyspace must be identified by a string that does not contain whitepace, while the shard is typically identified by a string in the format `<range start>-<range end>`.
- `<served tablet type>` – Required. The vttablet's role. Valid values are:

  - `backup` – A slaved copy of data that is offline to queries other than for backup purposes
  - `batch` – A slaved copy of data for OLAP load patterns (typically for MapReduce jobs)
  - `drained` – A tablet that is reserved for a background process. For example, a tablet used by a vtworker process, where the tablet is likely lagging in replication.
  - `experimental` – A slaved copy of data that is ready but not serving query traffic. The value indicates a special characteristic of the tablet that indicates the tablet should not be considered a potential master. Vitess also does not worry about lag for experimental tablets when reparenting.
  - `master` – A primary copy of data
  - `rdonly` – A slaved copy of data for OLAP load patterns
  - `replica` – A slaved copy of data ready to be promoted to master
  - `restore` – A tablet that is restoring from a snapshot. Typically, this happens at tablet startup, then it goes to its right state.
  - `schema_apply` – A slaved copy of data that had been serving query traffic but that is now applying a schema change. Following the change, the tablet will revert to its serving type.
  - `snapshot_source` – A slaved copy of data where mysqld is **not** running and where Vitess is serving data files to clone slaves. Use this command to enter this mode:

    ```
    vtctl Snapshot -server-mode ...
    ```

    Use this command to exit this mode:

    ```
    vtctl SnapshotSourceEnd ...
    ```
  - `spare` – A slaved copy of data that is ready but not serving query traffic. The data could be a potential master tablet.

#### Errors

- the `<keyspace/shard>` and `<served tablet type>` arguments are both required for the `<SetShardServedTypes>` command This error occurs if the command is not called with exactly 2 arguments.

### SetShardTabletControl

Sets the TabletControl record for a shard and type. Only use this for an emergency fix or after a finished vertical split. The *MigrateServedFrom* and *MigrateServedType* commands set this field appropriately already. Always specify the blacklisted\_tables flag for vertical splits, but never for horizontal splits.  
  
To set the DisableQueryServiceFlag, keep 'blacklisted\_tables' empty, and set 'disable\_query\_service' to true or false. Useful to fix horizontal splits gone wrong.  
  
To change the blacklisted tables list, specify the 'blacklisted\_tables' parameter with the new list. Useful to fix tables that are being blocked after a vertical split.  
  
To just remove the ShardTabletControl entirely, use the 'remove' flag, useful after a vertical split is finished to remove serving restrictions.

#### Example

```
SetShardTabletControl [--cells=c1,c2,...] [--blacklisted_tables=t1,t2,...] [--remove] [--disable_query_service] <keyspace/shard> <tablet type>
```

#### Flags

| Name | Type | Definition |
| --- | --- | --- |
| blacklisted\_tables | string | Specifies a comma-separated list of tables to blacklist (used for vertical split). Each is either an exact match, or a regular expression of the form '/regexp/'. |
| cells | string | Specifies a comma-separated list of cells to update |
| disable\_query\_service | Boolean | Disables query service on the provided nodes. This flag requires 'blacklisted\_tables' and 'remove' to be unset, otherwise it's ignored. |
| remove | Boolean | Removes cells for vertical splits. |

#### Arguments

- `<keyspace/shard>` – Required. The name of a sharded database that contains one or more tables as well as the shard associated with the command. The keyspace must be identified by a string that does not contain whitepace, while the shard is typically identified by a string in the format `<range start>-<range end>`.
- `<tablet type>` – Required. The vttablet's role. Valid values are:

  - `backup` – A slaved copy of data that is offline to queries other than for backup purposes
  - `batch` – A slaved copy of data for OLAP load patterns (typically for MapReduce jobs)
  - `drained` – A tablet that is reserved for a background process. For example, a tablet used by a vtworker process, where the tablet is likely lagging in replication.
  - `experimental` – A slaved copy of data that is ready but not serving query traffic. The value indicates a special characteristic of the tablet that indicates the tablet should not be considered a potential master. Vitess also does not worry about lag for experimental tablets when reparenting.
  - `master` – A primary copy of data
  - `rdonly` – A slaved copy of data for OLAP load patterns
  - `replica` – A slaved copy of data ready to be promoted to master
  - `restore` – A tablet that is restoring from a snapshot. Typically, this happens at tablet startup, then it goes to its right state.
  - `schema_apply` – A slaved copy of data that had been serving query traffic but that is now applying a schema change. Following the change, the tablet will revert to its serving type.
  - `snapshot_source` – A slaved copy of data where mysqld is **not** running and where Vitess is serving data files to clone slaves. Use this command to enter this mode:

    ```
    vtctl Snapshot -server-mode ...
    ```

    Use this command to exit this mode:

    ```
    vtctl SnapshotSourceEnd ...
    ```
  - `spare` – A slaved copy of data that is ready but not serving query traffic. The data could be a potential master tablet.

#### Errors

- the `<keyspace/shard>` and `<tablet type>` arguments are both required for the `<SetShardTabletControl>` command This error occurs if the command is not called with exactly 2 arguments.

### ShardReplicationFix

Walks through a ShardReplication object and fixes the first error that it encounters.

#### Example

```
ShardReplicationFix <cell> <keyspace/shard>
```

#### Arguments

- `<cell>` – Required. A cell is a location for a service. Generally, a cell resides in only one cluster. In Vitess, the terms "cell" and "data center" are interchangeable. The argument value is a string that does not contain whitespace.
- `<keyspace/shard>` – Required. The name of a sharded database that contains one or more tables as well as the shard associated with the command. The keyspace must be identified by a string that does not contain whitepace, while the shard is typically identified by a string in the format `<range start>-<range end>`.

#### Errors

- the `<cell>` and `<keyspace/shard>` arguments are required for the ShardReplicationRemove command This error occurs if the command is not called with exactly 2 arguments.

### ShardReplicationPositions

Shows the replication status of each slave machine in the shard graph. In this case, the status refers to the replication lag between the master vttablet and the slave vttablet. In Vitess, data is always written to the master vttablet first and then replicated to all slave vttablets. Output is sorted by tablet type, then replication position. Use ctrl-C to interrupt command and see partial result if needed.

#### Example

```
ShardReplicationPositions <keyspace/shard>
```

#### Arguments

- `<keyspace/shard>` – Required. The name of a sharded database that contains one or more tables as well as the shard associated with the command. The keyspace must be identified by a string that does not contain whitepace, while the shard is typically identified by a string in the format `<range start>-<range end>`.

#### Errors

- the `<keyspace/shard>` argument is required for the `<ShardReplicationPositions>` command This error occurs if the command is not called with exactly one argument.

### SourceShardAdd

Adds the SourceShard record with the provided index. This is meant as an emergency function. It does not call RefreshState for the shard master.

#### Example

```
SourceShardAdd [--key_range=<keyrange>] [--tables=<table1,table2,...>] <keyspace/shard> <uid> <source keyspace/shard>
```

#### Flags

| Name | Type | Definition |
| --- | --- | --- |
| key\_range | string | Identifies the key range to use for the SourceShard |
| tables | string | Specifies a comma-separated list of tables to replicate (used for vertical split). Each is either an exact match, or a regular expression of the form /regexp/ |

#### Arguments

- `<keyspace/shard>` – Required. The name of a sharded database that contains one or more tables as well as the shard associated with the command. The keyspace must be identified by a string that does not contain whitepace, while the shard is typically identified by a string in the format `<range start>-<range end>`.
- `<uid>` – Required.
- `<source keyspace/shard>` – Required. The name of a sharded database that contains one or more tables as well as the shard associated with the command. The keyspace must be identified by a string that does not contain whitepace, while the shard is typically identified by a string in the format `<range start>-<range end>`.

#### Errors

- the `<keyspace/shard>`, `<uid>`, and `<source keyspace/shard>` arguments are all required for the `<SourceShardAdd>` command This error occurs if the command is not called with exactly 3 arguments.

### SourceShardDelete

Deletes the SourceShard record with the provided index. This is meant as an emergency cleanup function. It does not call RefreshState for the shard master.

#### Example

```
SourceShardDelete <keyspace/shard> <uid>
```

#### Arguments

- `<keyspace/shard>` – Required. The name of a sharded database that contains one or more tables as well as the shard associated with the command. The keyspace must be identified by a string that does not contain whitepace, while the shard is typically identified by a string in the format `<range start>-<range end>`.
- `<uid>` – Required.

#### Errors

- the `<keyspace/shard>` and `<uid>` arguments are both required for the `<SourceShardDelete>` command This error occurs if the command is not called with at least 2 arguments.

### TabletExternallyReparented

Changes metadata in the topology server to acknowledge a shard master change performed by an external tool. See the Reparenting guide for more information:https://github.com/youtube/vitess/blob/master/doc/Reparenting.md#external-reparents.

#### Example

```
TabletExternallyReparented <tablet alias>
```

#### Arguments

- `<tablet alias>` – Required. A Tablet Alias uniquely identifies a vttablet. The argument value is in the format `<cell name>-<uid>`.

#### Errors

- the `<tablet alias>` argument is required for the `<TabletExternallyReparented>` command This error occurs if the command is not called with exactly one argument.

### ValidateShard

Validates that all nodes that are reachable from this shard are consistent.

#### Example

```
ValidateShard [-ping-tablets] <keyspace/shard>
```

#### Flags

| Name | Type | Definition |
| --- | --- | --- |
| ping-tablets | Boolean | Indicates whether all tablets should be pinged during the validation process |

#### Arguments

- `<keyspace/shard>` – Required. The name of a sharded database that contains one or more tables as well as the shard associated with the command. The keyspace must be identified by a string that does not contain whitepace, while the shard is typically identified by a string in the format `<range start>-<range end>`.

#### Errors

- the `<keyspace/shard>` argument is required for the `<ValidateShard>` command This error occurs if the command is not called with exactly one argument.

### WaitForFilteredReplication

Blocks until the specified shard has caught up with the filtered replication of its source shard.

#### Example

```
WaitForFilteredReplication [-max_delay <max_delay, default 30s>] <keyspace/shard>
```

#### Arguments

- `<keyspace/shard>` – Required. The name of a sharded database that contains one or more tables as well as the shard associated with the command. The keyspace must be identified by a string that does not contain whitepace, while the shard is typically identified by a string in the format `<range start>-<range end>`.

#### Errors

- the `<keyspace/shard>` argument is required for the `<WaitForFilteredReplication>` command This error occurs if the command is not called with exactly one argument.

## Tablets

- Backup
- ChangeSlaveType
- DeleteTablet
- ExecuteFetchAsDba
- ExecuteHook
- GetTablet
- IgnoreHealthError
- InitTablet
- Ping
- RefreshState
- RefreshStateByShard
- ReparentTablet
- RestoreFromBackup
- RunHealthCheck
- SetReadOnly
- SetReadWrite
- Sleep
- StartSlave
- StopSlave
- UpdateTabletAddrs

### Backup

Stops mysqld and uses the BackupStorage service to store a new backup. This function also remembers if the tablet was replicating so that it can restore the same state after the backup completes.

#### Example

```
Backup [-concurrency=4] <tablet alias>
```

#### Flags

| Name | Type | Definition |
| --- | --- | --- |
| concurrency | Int | Specifies the number of compression/checksum jobs to run simultaneously |

#### Arguments

- `<tablet alias>` – Required. A Tablet Alias uniquely identifies a vttablet. The argument value is in the format `<cell name>-<uid>`.

#### Errors

- the `<Backup>` command requires the `<tablet alias>` argument This error occurs if the command is not called with exactly one argument.

### ChangeSlaveType

Changes the db type for the specified tablet, if possible. This command is used primarily to arrange replicas, and it will not convert a master.  
  
NOTE: This command automatically updates the serving graph.

#### Example

```
ChangeSlaveType [-dry-run] <tablet alias> <tablet type>
```

#### Flags

| Name | Type | Definition |
| --- | --- | --- |
| dry-run | Boolean | Lists the proposed change without actually executing it |

#### Arguments

- `<tablet alias>` – Required. A Tablet Alias uniquely identifies a vttablet. The argument value is in the format `<cell name>-<uid>`.
- `<tablet type>` – Required. The vttablet's role. Valid values are:

  - `backup` – A slaved copy of data that is offline to queries other than for backup purposes
  - `batch` – A slaved copy of data for OLAP load patterns (typically for MapReduce jobs)
  - `drained` – A tablet that is reserved for a background process. For example, a tablet used by a vtworker process, where the tablet is likely lagging in replication.
  - `experimental` – A slaved copy of data that is ready but not serving query traffic. The value indicates a special characteristic of the tablet that indicates the tablet should not be considered a potential master. Vitess also does not worry about lag for experimental tablets when reparenting.
  - `master` – A primary copy of data
  - `rdonly` – A slaved copy of data for OLAP load patterns
  - `replica` – A slaved copy of data ready to be promoted to master
  - `restore` – A tablet that is restoring from a snapshot. Typically, this happens at tablet startup, then it goes to its right state.
  - `schema_apply` – A slaved copy of data that had been serving query traffic but that is now applying a schema change. Following the change, the tablet will revert to its serving type.
  - `snapshot_source` – A slaved copy of data where mysqld is **not** running and where Vitess is serving data files to clone slaves. Use this command to enter this mode:

    ```
    vtctl Snapshot -server-mode ...
    ```

    Use this command to exit this mode:

    ```
    vtctl SnapshotSourceEnd ...
    ```
  - `spare` – A slaved copy of data that is ready but not serving query traffic. The data could be a potential master tablet.

#### Errors

- the `<tablet alias>` and `<db type>` arguments are required for the `<ChangeSlaveType>` command This error occurs if the command is not called with exactly 2 arguments.
- failed reading tablet %v: %v
- invalid type transition %v: %v -> %v

### DeleteTablet

Deletes tablet(s) from the topology.

#### Example

```
DeleteTablet [-allow_master] <tablet alias> ...
```

#### Flags

| Name | Type | Definition |
| --- | --- | --- |
| allow\_master | Boolean | Allows for the master tablet of a shard to be deleted. Use with caution. |

#### Arguments

- `<tablet alias>` – Required. A Tablet Alias uniquely identifies a vttablet. The argument value is in the format `<cell name>-<uid>`. To specify multiple values for this argument, separate individual values with a space.

#### Errors

- the `<tablet alias>` argument must be used to specify at least one tablet when calling the `<DeleteTablet>` command This error occurs if the command is not called with at least one argument.

### ExecuteFetchAsDba

Runs the given SQL command as a DBA on the remote tablet.

#### Example

```
ExecuteFetchAsDba [-max_rows=10000] [-disable_binlogs] [-json] <tablet alias> <sql command>
```

#### Flags

| Name | Type | Definition |
| --- | --- | --- |
| disable\_binlogs | Boolean | Disables writing to binlogs during the query |
| json | Boolean | Output JSON instead of human-readable table |
| max\_rows | Int | Specifies the maximum number of rows to allow in reset |
| reload\_schema | Boolean | Indicates whether the tablet schema will be reloaded after executing the SQL command. The default value is `false`, which indicates that the tablet schema will not be reloaded. |

#### Arguments

- `<tablet alias>` – Required. A Tablet Alias uniquely identifies a vttablet. The argument value is in the format `<cell name>-<uid>`.
- `<sql command>` – Required.

#### Errors

- the `<tablet alias>` and `<sql command>` arguments are required for the `<ExecuteFetchAsDba>` command This error occurs if the command is not called with exactly 2 arguments.

### ExecuteHook

Runs the specified hook on the given tablet. A hook is a script that resides in the $VTROOT/vthook directory. You can put any script into that directory and use this command to run that script.  
  
For this command, the param=value arguments are parameters that the command passes to the specified hook.

#### Example

```
ExecuteHook <tablet alias> <hook name> [<param1=value1> <param2=value2> ...]
```

#### Arguments

- `<tablet alias>` – Required. A Tablet Alias uniquely identifies a vttablet. The argument value is in the format `<cell name>-<uid>`.
- `<hook name>` – Required.
- `<param1=value1>` `<param2=value2>` . – Optional.

#### Errors

- the `<tablet alias>` and `<hook name>` arguments are required for the `<ExecuteHook>` command This error occurs if the command is not called with at least 2 arguments.

### GetTablet

Outputs a JSON structure that contains information about the Tablet.

#### Example

```
GetTablet <tablet alias>
```

#### Arguments

- `<tablet alias>` – Required. A Tablet Alias uniquely identifies a vttablet. The argument value is in the format `<cell name>-<uid>`.

#### Errors

- the `<tablet alias>` argument is required for the `<GetTablet>` command This error occurs if the command is not called with exactly one argument.

### IgnoreHealthError

Sets the regexp for health check errors to ignore on the specified tablet. The pattern has implicit ^$ anchors. Set to empty string or restart vttablet to stop ignoring anything.

#### Example

```
IgnoreHealthError <tablet alias> <ignore regexp>
```

#### Arguments

- `<tablet alias>` – Required. A Tablet Alias uniquely identifies a vttablet. The argument value is in the format `<cell name>-<uid>`.
- `<ignore regexp>` – Required.

#### Errors

- the `<tablet alias>` and `<ignore regexp>` arguments are required for the `<IgnoreHealthError>` command This error occurs if the command is not called with exactly 2 arguments.

### InitTablet

Initializes a tablet in the topology.

#### Example

```
InitTablet [-allow_update] [-allow_different_shard] [-allow_master_override] [-parent] [-db_name_override=<db name>] [-hostname=<hostname>] [-mysql_port=<port>] [-port=<port>] [-grpc_port=<port>] -keyspace=<keyspace> -shard=<shard> <tablet alias> <tablet type>
```

#### Flags

| Name | Type | Definition |
| --- | --- | --- |
| allow\_master\_override | Boolean | Use this flag to force initialization if a tablet is created as master, and a master for the keyspace/shard already exists. Use with caution. |
| allow\_update | Boolean | Use this flag to force initialization if a tablet with the same name already exists. Use with caution. |
| db\_name\_override | string | Overrides the name of the database that the vttablet uses |
| grpc\_port | Int | The gRPC port for the vttablet process |
| hostname | string | The server on which the tablet is running |
| keyspace | string | The keyspace to which this tablet belongs |
| mysql\_port | Int | The mysql port for the mysql daemon |
| parent | Boolean | Creates the parent shard and keyspace if they don't yet exist |
| port | Int | The main port for the vttablet process |
| shard | string | The shard to which this tablet belongs |
| tags | string | A comma-separated list of key:value pairs that are used to tag the tablet |

#### Arguments

- `<tablet alias>` – Required. A Tablet Alias uniquely identifies a vttablet. The argument value is in the format `<cell name>-<uid>`.
- `<tablet type>` – Required. The vttablet's role. Valid values are:

  - `backup` – A slaved copy of data that is offline to queries other than for backup purposes
  - `batch` – A slaved copy of data for OLAP load patterns (typically for MapReduce jobs)
  - `drained` – A tablet that is reserved for a background process. For example, a tablet used by a vtworker process, where the tablet is likely lagging in replication.
  - `experimental` – A slaved copy of data that is ready but not serving query traffic. The value indicates a special characteristic of the tablet that indicates the tablet should not be considered a potential master. Vitess also does not worry about lag for experimental tablets when reparenting.
  - `master` – A primary copy of data
  - `rdonly` – A slaved copy of data for OLAP load patterns
  - `replica` – A slaved copy of data ready to be promoted to master
  - `restore` – A tablet that is restoring from a snapshot. Typically, this happens at tablet startup, then it goes to its right state.
  - `schema_apply` – A slaved copy of data that had been serving query traffic but that is now applying a schema change. Following the change, the tablet will revert to its serving type.
  - `snapshot_source` – A slaved copy of data where mysqld is **not** running and where Vitess is serving data files to clone slaves. Use this command to enter this mode:

    ```
    vtctl Snapshot -server-mode ...
    ```

    Use this command to exit this mode:

    ```
    vtctl SnapshotSourceEnd ...
    ```
  - `spare` – A slaved copy of data that is ready but not serving query traffic. The data could be a potential master tablet.

#### Errors

- the `<tablet alias>` and `<tablet type>` arguments are both required for the `<InitTablet>` command This error occurs if the command is not called with exactly 2 arguments.

### Ping

Checks that the specified tablet is awake and responding to RPCs. This command can be blocked by other in-flight operations.

#### Example

```
Ping <tablet alias>
```

#### Arguments

- `<tablet alias>` – Required. A Tablet Alias uniquely identifies a vttablet. The argument value is in the format `<cell name>-<uid>`.

#### Errors

- the `<tablet alias>` argument is required for the `<Ping>` command This error occurs if the command is not called with exactly one argument.

### RefreshState

Reloads the tablet record on the specified tablet.

#### Example

```
RefreshState <tablet alias>
```

#### Arguments

- `<tablet alias>` – Required. A Tablet Alias uniquely identifies a vttablet. The argument value is in the format `<cell name>-<uid>`.

#### Errors

- the `<tablet alias>` argument is required for the `<RefreshState>` command This error occurs if the command is not called with exactly one argument.

### RefreshStateByShard

Runs 'RefreshState' on all tablets in the given shard.

#### Example

```
RefreshStateByShard [-cells=c1,c2,...] <keyspace/shard>
```

#### Flags

| Name | Type | Definition |
| --- | --- | --- |
| cells | string | Specifies a comma-separated list of cells whose tablets are included. If empty, all cells are considered. |

#### Arguments

- `<keyspace/shard>` – Required. The name of a sharded database that contains one or more tables as well as the shard associated with the command. The keyspace must be identified by a string that does not contain whitepace, while the shard is typically identified by a string in the format `<range start>-<range end>`.

#### Errors

- the `<keyspace/shard>` argument is required for the `<RefreshStateByShard>` command This error occurs if the command is not called with exactly one argument.

### ReparentTablet

Reparent a tablet to the current master in the shard. This only works if the current slave position matches the last known reparent action.

#### Example

```
ReparentTablet <tablet alias>
```

#### Errors

- action `<ReparentTablet>` requires `<tablet alias>` This error occurs if the command is not called with exactly one argument.

### RestoreFromBackup

Stops mysqld and restores the data from the latest backup.

#### Example

```
RestoreFromBackup <tablet alias>
```

#### Errors

- the `<RestoreFromBackup>` command requires the `<tablet alias>` argument This error occurs if the command is not called with exactly one argument.

### RunHealthCheck

Runs a health check on a remote tablet.

#### Example

```
RunHealthCheck <tablet alias>
```

#### Arguments

- `<tablet alias>` – Required. A Tablet Alias uniquely identifies a vttablet. The argument value is in the format `<cell name>-<uid>`.

#### Errors

- the `<tablet alias>` argument is required for the `<RunHealthCheck>` command This error occurs if the command is not called with exactly one argument.

### SetReadOnly

Sets the tablet as read-only.

#### Example

```
SetReadOnly <tablet alias>
```

#### Arguments

- `<tablet alias>` – Required. A Tablet Alias uniquely identifies a vttablet. The argument value is in the format `<cell name>-<uid>`.

#### Errors

- the `<tablet alias>` argument is required for the `<SetReadOnly>` command This error occurs if the command is not called with exactly one argument.
- failed reading tablet %v: %v

### SetReadWrite

Sets the tablet as read-write.

#### Example

```
SetReadWrite <tablet alias>
```

#### Arguments

- `<tablet alias>` – Required. A Tablet Alias uniquely identifies a vttablet. The argument value is in the format `<cell name>-<uid>`.

#### Errors

- the `<tablet alias>` argument is required for the `<SetReadWrite>` command This error occurs if the command is not called with exactly one argument.
- failed reading tablet %v: %v

### Sleep

Blocks the action queue on the specified tablet for the specified amount of time. This is typically used for testing.

#### Example

```
Sleep <tablet alias> <duration>
```

#### Arguments

- `<tablet alias>` – Required. A Tablet Alias uniquely identifies a vttablet. The argument value is in the format `<cell name>-<uid>`.
- `<duration>` – Required. The amount of time that the action queue should be blocked. The value is a string that contains a possibly signed sequence of decimal numbers, each with optional fraction and a unit suffix, such as "300ms" or "1h45m". See the definition of the Go language's ParseDuration function for more details. Note that, in practice, the value should be a positively signed value.

#### Errors

- the `<tablet alias>` and `<duration>` arguments are required for the `<Sleep>` command This error occurs if the command is not called with exactly 2 arguments.

### StartSlave

Starts replication on the specified slave.

#### Example

```
StartSlave <tablet alias>
```

#### Arguments

- `<tablet alias>` – Required. A Tablet Alias uniquely identifies a vttablet. The argument value is in the format `<cell name>-<uid>`.

#### Errors

- action `<StartSlave>` requires `<tablet alias>` This error occurs if the command is not called with exactly one argument.
- failed reading tablet %v: %v

### StopSlave

Stops replication on the specified slave.

#### Example

```
StopSlave <tablet alias>
```

#### Arguments

- `<tablet alias>` – Required. A Tablet Alias uniquely identifies a vttablet. The argument value is in the format `<cell name>-<uid>`.

#### Errors

- action `<StopSlave>` requires `<tablet alias>` This error occurs if the command is not called with exactly one argument.
- failed reading tablet %v: %v

### UpdateTabletAddrs

Updates the IP address and port numbers of a tablet.

#### Example

```
UpdateTabletAddrs [-hostname <hostname>] [-ip-addr <ip addr>] [-mysql-port <mysql port>] [-vt-port <vt port>] [-grpc-port <grpc port>] <tablet alias>
```

#### Flags

| Name | Type | Definition |
| --- | --- | --- |
| grpc-port | Int | The gRPC port for the vttablet process |
| hostname | string | The fully qualified host name of the server on which the tablet is running. |
| ip-addr | string | IP address |
| mysql-port | Int | The mysql port for the mysql daemon |
| vt-port | Int | The main port for the vttablet process |

#### Arguments

- `<tablet alias>` – Required. A Tablet Alias uniquely identifies a vttablet. The argument value is in the format `<cell name>-<uid>`.

#### Errors

- the `<tablet alias>` argument is required for the `<UpdateTabletAddrs>` command This error occurs if the command is not called with exactly one argument.
- malformed address: %v

## Workflows

- WorkflowAction
- WorkflowCreate
- WorkflowDelete
- WorkflowStart
- WorkflowStop
- WorkflowTree
- WorkflowWait

### WorkflowAction

Sends the provided action name on the specified path.

#### Example

```
WorkflowAction <path> <name>
```

#### Arguments

- `<name>` – Required.

#### Errors

- the `<path>` and `<name>` arguments are required for the `<WorkflowAction>` command This error occurs if the command is not called with exactly 2 arguments.
- no workflow.Manager registered

### WorkflowCreate

Creates the workflow with the provided parameters. The workflow is also started, unless -skip\_start is specified.

#### Example

```
WorkflowCreate [-skip_start] <factoryName> [parameters...]
```

#### Flags

| Name | Type | Definition |
| --- | --- | --- |
| skip\_start | Boolean | If set, the workflow will not be started. |

#### Arguments

- `<factoryName>` – Required.

#### Errors

- the `<factoryName>` argument is required for the `<WorkflowCreate>` command This error occurs if the command is not called with at least one argument.
- no workflow.Manager registered

### WorkflowDelete

Deletes the finished or not started workflow.

#### Example

```
WorkflowDelete <uuid>
```

#### Errors

- the `<uuid>` argument is required for the `<WorkflowDelete>` command This error occurs if the command is not called with exactly one argument.
- no workflow.Manager registered

### WorkflowStart

Starts the workflow.

#### Example

```
WorkflowStart <uuid>
```

#### Errors

- the `<uuid>` argument is required for the `<WorkflowStart>` command This error occurs if the command is not called with exactly one argument.
- no workflow.Manager registered

### WorkflowStop

Stops the workflow.

#### Example

```
WorkflowStop <uuid>
```

#### Errors

- the `<uuid>` argument is required for the `<WorkflowStop>` command This error occurs if the command is not called with exactly one argument.
- no workflow.Manager registered

### WorkflowTree

Displays a JSON representation of the workflow tree.

#### Example

```
WorkflowTree
```

#### Errors

- the `<WorkflowTree>` command takes no parameter This error occurs if the command is not called with exactly 0 arguments.
- no workflow.Manager registered

### WorkflowWait

Waits for the workflow to finish.

#### Example

```
WorkflowWait <uuid>
```

#### Errors

- the `<uuid>` argument is required for the `<WorkflowWait>` command This error occurs if the command is not called with exactly one argument.
- no workflow.Manager registered

Contact: vitess@googlegroups.com  **·**  
Announcements  **·**  
© 2017 Vitess powered by Google Inc
